# Parental and School Influences on Character Attributes Among Chinese Adolescents

**DOI:** 10.3389/fped.2022.817471

**Published:** 2022-02-25

**Authors:** Xiaoqin Zhu, Daniel T. L. Shek, Lu Yu

**Affiliations:** Department of Applied Social Sciences, The Hong Kong Polytechnic University, Hong Kong, Hong Kong SAR, China

**Keywords:** character, social influence, ecological assets, parenting, youth development

## Abstract

**Objectives:**

While there are many theoretical propositions on parental and school impacts on character formation among adolescents, limited empirical evidence is available. To fill the research gaps, this study examined how parental factors (e.g., behavioral control, support, parent–child relationship, and psychological control) and school factors (teacher–student relationship and student–student relationship) affect adolescents' character attributes in the areas of “treating others well,” “self-discipline and law-abidance,” and “traditional Chinese virtues,” from both child and parent perspectives.

**Methods:**

We used survey data collected from 2,472 Chinese adolescents (Mean age = 14.76 ± 1.82 years; 1,271 girls) and 412 Chinese parents (Mean age = 45.68 ± 6.53 years; 313 mothers) in Hong Kong.

**Results:**

Structural equation modeling revealed significant positive effects of positive parental factors and relational factors in school on children's character attributes. However, psychological control as the indicator of negative parenting did not serve as a negative predictor of adolescents' character attributes.

**Conclusion:**

These findings shed light on the unique contribution of different parental and school factors to character development among Chinese adolescents. The findings also imply the importance of promoting ecological assets in family and school through parent and teacher training in effective character education, which is essential for children and adolescents' healthy development.

## Introduction

In view of the growing mental health issues among adolescents (1, 2), the past several decades have witnessed an increasing emphasis on the importance of promoting healthy youth development (3, 4). As an important indicator of healthy youth functioning, the development of character attributes is closely associated with a better quality of life indexed by lower psychological morbidity, fewer emotional and behavioral problems, better school engagement and achievement, and higher life satisfaction and happiness (5–10).

Character and temperament have been conceived as two major elements of personality in describing individual differences in ways of thinking, feeling, and behaving (11, 12). Temperament reflects features configuring automatic behavioral responses that are largely determined by biological factors (e.g., sensory processing and neurotransmitter systems) and is relatively less affected by learning and environmental factors, whereas character is regarded as higher functions that are oriented by temperament and develop during the ontogenetic with a major contribution of individual learning and social influences (13, 14). Historically, “personality” or “temperament” have been treated as morally “neutral” concepts and become the major focus of personality studies while the character is used to describe morally valued (or positive) personality attributes and draws much attention since the development of positive psychology (7). Given the positive nature of the character, other terms, such as virtue, moral character, moral trait, and character strength, are also used when referring to the positive personality attributes (6, 10).

Notably, different frameworks have been proposed to conceptualize character. For example, nine key moral traits were considered central to an individual's moral identity (15), including “kindness,” “compassion,” “care,” “friendliness,” “generosity,” “helpfulness,” “fairness,” “diligence,” and “honesty.” The list was enriched by Hardy et al. (16) to include 20 character attributes, such as “generosity,” “respect,” “loyalty,” “responsibility,” and “consideration.” Based on positive psychology perspectives, Peterson and Seligman (6) proposed the “values-in-action” (VIA) classification, which consists of six virtues underpinned by 24 character strengths, to summarize a comprehensive framework of character attributes that are universally valued. Despite different terminologies (e.g., virtue, moral character, character strength, etc.) used in these conceptualizations, a consensus is that character attributes are morally valued personality traits. They guide appropriate motivation and behavior with a concern for the good of others and society, which is intrinsically conducive to, and even constitutive of, a happy and good life (7, 17).

Given the significance of character attributes in adolescent development, a question that merits investigation is what factors contribute to the development of character attributes in adolescents. The developmental systems framework highlights that individual traits are shaped by interactions between the child and his or her living environments at different levels, such as family, school, society, and culture (18). According to this view, character attributes as positive personality traits are susceptible to the influence of systemic relations between children and their social world (19–22). For instance, Park (22) proposed five factors that may contribute to character development among children and adolescents. These factors include biological factors, positive role models, parenting styles, close relationships with friends and family members, and other positive institutions, among which the latter four factors represent social influence in nature. Similarly, Narvaez and Lapsley (21) noted that “moral character is shaped by multiple levels of social influence including caring relationships, cultural climates, and a supportive community in a type of moral ecological context” (p. 231).

Among different social factors, family and school-related factors especially deserve attention. Narvaez and Lapsley (21) commented that “caregivers, usually parents, have a great deal of influence on moral development” (p. 238). It is argued that the first step of moral development is exposure to and internalization of moral values and norms, such as attitudes and standards of behavior modeled after others in social contexts (23). Family is the primary social context where parents not only communicate norms, standards, and values but also reinforce them in daily parenting practices, such as parental support, care, conversation, discipline, interrogation, emphasis, and punishment (21). Meanwhile, the child integrates the socio-emotional patterns in routine parent–child interactions into his or her autobiographical memory, based on which the child forms general expectations for the social life and builds moral representations of life. For example, children learn to care for others based on consistent warm responses and care from parents as well as secure attachment to parents (24). In general, positive parenting (e.g., support, warmth, responsiveness, consistent discipline, etc.) is expected to result in a successful socialization process that facilitates children to internalize moral norms and develop a moral sense of what is right or wrong. In contrast, negative parenting (e.g., overprotection, rejection, etc.) is more likely to hinder the internalization process and moral development in children (25, 26).

Apart from the family, students spend a large amount of time in school, thus making the school an important ecological system in fostering character development. While parents are the primary socialization agents who establish the foundation for the child's character development, the school serves as the secondary socialization agent which consolidates or adjusts this process. Indeed, the World Health Organization (27) highlighted the importance of schooling and noted that “among all the sectors that play critical roles in adolescent health, education is key” (p. 8). Continuous exposure to ecological assets in the school context, such as teacher support, positive peer interactions, and positive school climate, provides students with everyday opportunities to learn and practice attitudes, skills, norms, and values, which consolidate and refine the development of character attributes (25). There has been an agreement in both theoretical propositions and empirical findings that effective character education in the school context needs to create a nurturing environment in addition to cultivating students' moral judgment or reasoning skills (28–30).

To sum up, family and school can be considered two “positive institutions,” which “facilitate the development and display of positive traits” [(31), p. 20]. It is believed that positive relationships and supportive environments in both family and school enhance adolescents' intrinsic motivation to develop internal moral codes and to internalize prosocial values and behave accordingly, which subsequently reinforce their character attributes (21, 25). Despite the plentiful theoretical discussion about parental and school influence on adolescents' character development, surprisingly very little empirical evidence is available. For example, we only identified 18 empirical studies published in English or Chinese when we searched the literature in May 2021 on the association between different parental factors and children's character attributes in related databases (e.g., PsychINFO, Embase, Web of Science, Scopus, and Chinese databases). As remarked by Lerner and Callina (19), “there is more conceptual consensus than empirical support for these ideas” (p. 322).

Among the few empirical studies in this area, their findings generally echo the existing theoretical propositions. For example, Ngai (32) and Ngai et al. (33) reported that parental care positively predicted character strengths (e.g., bravery, perseverance, kindness, and self-regulation) among adolescents, whereas over-controlling parenting exerted negative influence. Similarly, Raimundi et al. (34) found that positive family functioning in terms of effective parent–child communication and family cohesion was positively associated with the development of character strengths in adolescent athletes. For school impacts, Shubert (35) found that school-related ecological assets such as teacher support and positive social relations formed in school were positive predictors of students' three positive character attributes, including teamwork, perseverance, and future orientation. Similarly, Carretero and Gimeno (36) reported that different dimensions of school climate significantly predicted students' character attributes.

Nevertheless, to our best knowledge, no studies have investigated both parental and school impacts simultaneously and shed light on their unique influence on adolescent character attributes. Furthermore, existing literature mainly utilized adolescents' self-report data without considering parents' perspectives. To fill these research gaps and provide additional empirical evidence for the association between parental and school factors and adolescents' character attributes, the present study aimed to investigate how positive and negative parenting, as well as teacher–student and student–student relationships, predict character attributes among adolescents. We attempted to address the following two research questions in this study.

Research Question 1: Are parental factors associated with character attributes in adolescents? It was expected that positive parenting practice would be positively associated with adolescents' character attributes (Hypothesis 1a). In contrast, negative parenting practice would be negatively associated with adolescents' character attributes (Hypothesis 1b).

Research Question 2: Are relational factors in the school associated with adolescent character attributes? It was hypothesized that teacher–student relationship (Hypothesis 2a) and student–student relationship (Hypothesis 2b) would be positively related to character attributes among adolescents.

The above hypotheses were tested in two studies. Study 1 tested Hypotheses 1a, 2a, 2b (i.e., positive parenting and relational factors in school) from a student perspective. Study 2 tested Hypotheses 1a and 1b (i.e., positive and negative parenting) from a parent perspective, aiming to replicate the findings in Study 1 based on the parent perspective and also to extend the investigation of parental factors from positive parenting to negative parenting.

## Study 1: Findings Based on Student Perspective

### Methods

#### Participants and Procedures

In 2017, a project entitled “Character building—A shared mission for a better future” was launched in Hong Kong to understand the development of character attributes and other psychosocial competencies among Chinese secondary school students. The project collected data from students, teachers, and parents on multiple aspects, such as adolescent character attributes and prosocial behaviors, parenting, relationship in school, and adolescent wellbeing (37). During data collection, all participants were informed of the principles of anonymity, confidentiality, voluntary participation, and free withdrawal. This project obtained ethical approval from the “Human Subjects Ethics Sub-Committee” in the authors' university.

This study utilized student data. The research team randomly selected 20 local secondary schools to participate in the project. Each participating school then randomly selected one or more classes of students and invited them to complete a student survey measuring their character attributes and other variables (38). Written consent was obtained from the schools, selected students, and their parents before data collection. During data collection, students were given sufficient time to respond to the questionnaires in their classroom settings with the presence of research staff.

A total of 2,474 students completed the survey. Among these student participants, 1,468 were from grade 7 to grade 9 (i.e., junior secondary stage) and 988 from grade 10 to grade 12 (i.e., senior secondary stage). The average age of the student participants was 14.76 years (SD = 1.82), and there were 1,271 girls. The majority of the participants reported living in intact families (*n* = 1,785, 72.2%) and without family economic disadvantage (*n* = 1,738, 70.3%). According to the territory-wide data in 2016, 17.8% of Hong Kong families with children were non-intact families (39) and 23.1% of children lived in low-income families (40). These figures suggest that the present sample was comparable to the related characteristics in the general population.

#### Measures

*Character Attributes* was measured by the 25-item “Personal Moral Character Scale” (PMCS), which was developed by the research team with reference to the existing measures of character. First, nine items (e.g., “kindness,” “compassion,” and “helpfulness”) were derived from Aquino and Reed's (15) list of moral attributes that are key to one's moral identity. This scale was validated and used in Chinese culture [e.g., (41)]. Second, 10 items (e.g., “respecting others,” “dedicated,” and “self-disciplined”) were selected from the Moral Values Scale, which was developed by Chen (42) and successfully employed in other research [e.g., (43)] in the Chinese context. The final six items (e.g., “loyalty,” “responsibility,” and “forgiving”) were adapted from the “Schwartz Values Survey” (44) and deemed morally valued in the Chinese context. Respondents evaluated their character attributes by reporting the extent to which they possess each attribute on a 5-point scale (1 = “very much unlike me,” 5 = “very much like me”). Exploratory factor analysis (EFA) and confirmatory factor analysis (CFA), each using one of the two half-samples, suggested a three-dimensional structure of these items. The first dimension included 10 character attributes intrinsic to “treating others well” (e.g., “friendly,” “generous,” “helpful,” and “compassion”). The second dimension pointed to “self-discipline and law abidance,” including eight attributes such as “hard-working,” “honest,” “law-abidance,” and “self-discipline.” The final dimension consisted of seven “traditional Chinese virtues,” such as “polite,” “loyalty,” and “respect for the elderly.” The details of the scale dimensionality have been reported elsewhere (10). In this study, both Cronbach's α and McDonald's ω (i.e., 0.83–0.89) indicated adequate internal consistency of the three dimensions (see Table 1).

**Table 1:** Reliabilities, descriptions, and correlations among variables in Study 1 (student data).

**Measures**	**Reliability (α/ω)**	***M***	***SD***	**Correlations**													
				**1**	**2**	**3**	**4**	**5**	**6**	**7**	**8**	**9**	**10**	**11**	**12**	**13**	**14**
Age		14.76	1.82	–													
Gender^a^				0.01	–												
FINT^b^				0.09^***^	0.06^**^	–											
FES^c^				−0.05^*^	0.05^*^	0.19^**^	–										
MCF1	0.89/0.89	3.84	0.58	0.01	0.12^***^	0.03	−0.01	–									
MCF2	0.84/0.84	3.56	0.60	−0.01	0.09^***^	0.08^***^	−0.03	0.71^***^	–								
MCF3	0.83/0.83	3.67	0.61	0.06^**^	0.11^***^	0.04	−0.001	0.69^***^	0.67^***^	–							
FBC	0.88/0.88	2.39	0.64	−0.11^***^	−0.02	0.17^***^	0.06^*^	0.19^***^	0.20^***^	0.21^***^	–						
FSU	0.87/0.87	2.82	0.78	−0.03	0.01	0.15^***^	0.08^**^	0.23^***^	0.18^***^	0.20^***^	0.60^***^	–					
FCR	0.87/0.89	2.57	0.81	−0.09^***^	0.03	0.18^***^	0.04	0.21^***^	0.20^***^	0.20^***^	0.64^***^	0.68^***^	–				
MBC	0.89/0.89	2.81	0.64	−0.13^***^	0.04	0.12^***^	0.04	0.31^***^	0.27^***^	0.27^***^	0.43^***^	0.27^***^	0.29^***^	–			
MSU	0.90/0.90	3.05	0.72	−0.06^**^	0.01	0.13^***^	0.07^*^	0.27^***^	0.23^***^	0.24^***^	0.26^***^	0.40^***^	0.30^***^	0.62^***^	–		
MCR	0.87/0.89	2.88	0.78	−0.07^**^	0.09^***^	0.12^***^	0.03	0.28^***^	0.26^***^	0.27^***^	0.30^***^	0.28^***^	0.43^***^	0.61^***^	0.72^***^	–	
TSR	0.88/0.88	2.90	0.56	0.01	−0.04	−0.01	−0.03	0.20^***^	0.17^***^	0.20^***^	0.16^***^	0.21^***^	0.17^***^	0.18^***^	0.18^***^	0.15^***^	–
SSR	0.91/0.91	2.93	0.58	0.01	0.003	0.07^**^	0.001	0.24^***^	0.19^***^	0.25^***^	0.19^***^	0.18^***^	0.21^***^	0.21^***^	0.19^***^	0.23^***^	0.40^***^

*FINT, family intactness; FES, family economic status; MCF1, moral character factor 1 (i.e., treating others well); MCF2, moral character factor 2 (i.e., self-discipline and law-abidance); MCF3, moral character factor 3 (i.e., traditional Chinese virtues); FBC, father's behavioral control; FSU, father's support; FCR, father–child relationship; MBC, mother's behavioral control; MSU, mother's support; MCR, mother–child relationship; TSR, teacher–student relationship; SSR, student–student relationship*.

a *1 = male; 2 = female*.

b *1 = non-intact family; 2 = intact family*.

c *1 = having family economic disadvantage; 2 = without family economic disadvantage*.

* *p < 0.05;*

** *p < 0.01;*

*** *p < 0.001*.

*Parental Factors* included three positive parenting indicators: behavioral control, support, and parent–child relationship. These factors were measured by respective subscales in the parenting scale developed by Shek and his collaborators (45, 46). These scales have been widely used to assess parenting practices in the Chinese context (47–49). The behavioral control subscale included seven items related to parental knowledge (e.g., “my father/mother knows my school life”), expectations (e.g., “my father/mother expects me to behave in a good manner in school”), and monitoring (e.g., “my father/mother actively understands my afterschool activities”). The parental support subscale contained three items, such as “my father/mother helps me when I need it.” The parent–child relationship subscale also consisted of three items, such as “I proactively share my feelings with my father/mother.” The participants reported their perceived parental practices on each item for their father and mother separately, on a 4-point scale (1 = “strongly disagree,” 4 = “strongly agree”). CFA showed that each subscale had a unidimensional structure in the present study. Cronbach's α and McDonald's ω (i.e., 0.87–0.90) indicated adequate reliability of the three dimensions for both paternal and maternal subscales (see Table 1).

*Relational Factors in School* included teacher–student and student–student relationships, measured by the two respective subscales in the validated Chinese version of Delaware School Climate Survey–Student (50, 51). Four items (e.g., “teachers care about students”) measured teacher–student relationship, and another four items (e.g., “students are friendly with each other”) assessed student–student relationship. The student participants gave their ratings on a 4-point scale (1 = “strongly disagree,” 4 = “strongly agree”). The two subscales were unidimensional and demonstrated adequate internal consistency in this study, with Cronbach's α and McDonald's ω above 0.85 (i.e., 0.88–0.91) (see Table 1).

*Covariates* included four variables, namely age, gender, family intactness, and family economic status that have been commonly controlled in previous studies (10, 49). Those students with parents in the first marriage were considered living in intact families while non-intactness was indexed by other types of parental marital status (e.g., re-married, divorced, or separated). Dependence on governmental welfare was used to indicate family economic disadvantage.

#### Data Analysis Plan

Preliminary analyses were conducted to check the multivariate normality of observed items in key measures (character attributes, parental factors, and relational factors in school), the dimensionality of each subscale, cluster effects of variables, and common method biases. Results indicated that all items were normally distributed with the absolute values of skewness and kurtosis below two and seven, respectively. Thus, the maximum likelihood estimation method (ML) was employed in the subsequent analyses (52). CFA indicated a three-dimensional structure of character attributes (10) and a unidimensional structure of other subscales. School-level intra-class correlation (ICC) for related variables and items of character attributes, parental factors, and relational factors in school varied between 0.0001 and 0.026, suggesting that very low proportions in students' responses to these measures were attributable to school-level cluster effects (53). Thus, there was no need to use multi-level modeling in the present study. To statistically detect common method biases (CMB), Harman's single-factor test was performed through exploratory factor analysis. It was revealed that only 24.19% of the variance in all the key measures were explained by a single factor, suggesting CMB was not a major issue as no one general factor would account for the major covariance among the measures (54).

**Figure 1:**
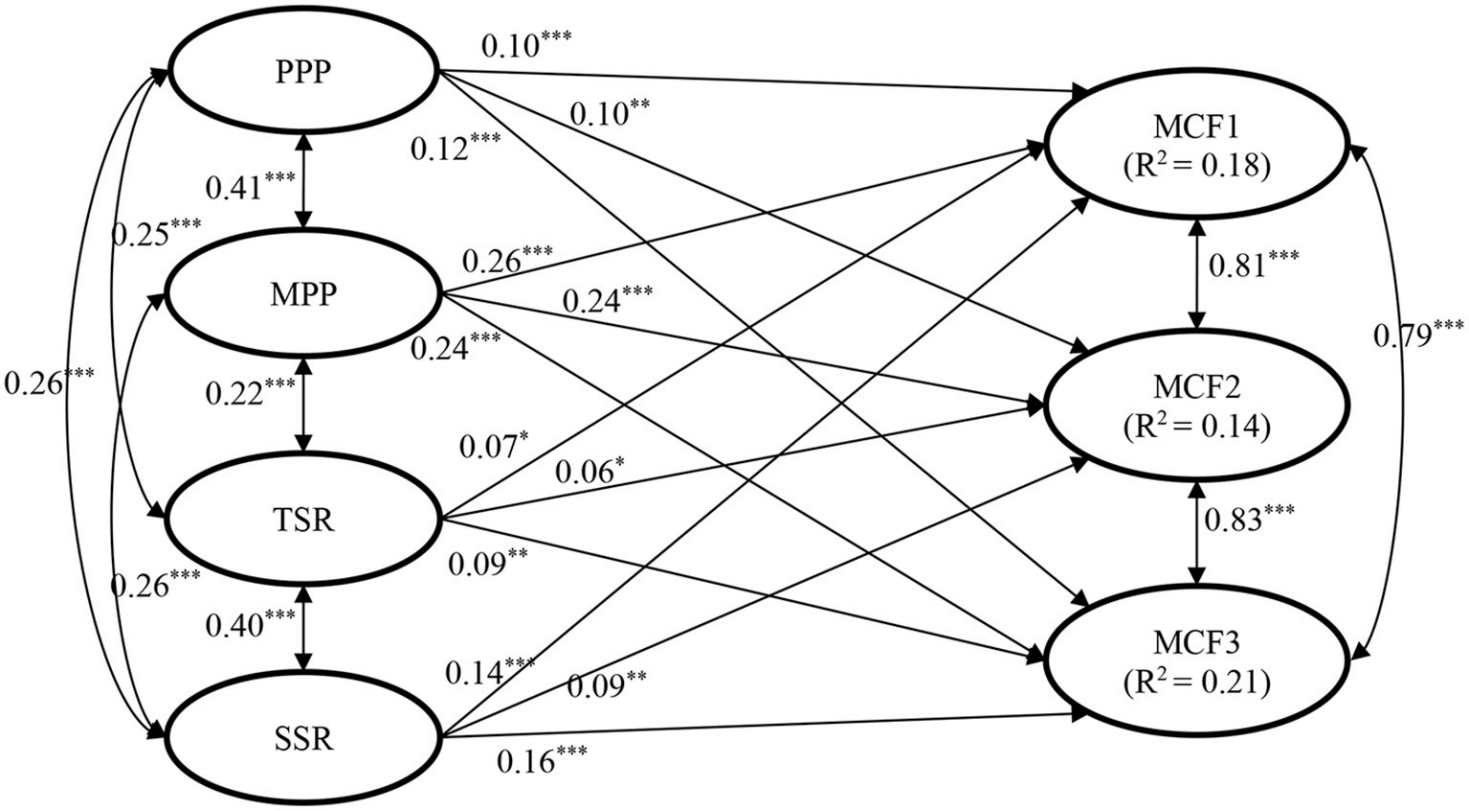
Standardized results of structural equation modeling on the relationships between parental and school factors and adolescent moral character in Study 1. Age, gender, family intactness, and family economic status were controlled. The control variables, observed indicators, and residuals were not shown in the figure. PPP, paternal positive parenting; MPP, maternal positive parenting; TSR, teacher–student relationship; SSR, student–student relationship; MCF1, moral character factor 1 (i.e., treating others well); MCF2, moral character factor 2 (i.e., self-discipline and law-abidance); MCF3, moral character factor 3 (i.e., traditional Chinese virtues). χ^2^ = 3559.789, df = 819, χ^2^/df = 4.34, CFI = 0.924, NNFI = 0.917, RMSEA = 0.041, SRMR = 0.037. *p < 0.05; **p < 0.01; ***p < 0.001.

Structural equation modeling (SEM) was performed using Mplus 8.5 to test the hypothesized predictions of parental and school factors (Figure 1 shows the model with regression coefficients). The “full information maximum likelihood estimation” that makes use of all available data of each participant was employed to deal with any missing values at the variable level. In SEM, there were four latent predictors, including (1) paternal positive parenting indicated by paternal behavioral control, paternal support, and father–child relationship; (2) maternal positive parenting indicated by maternal behavioral control, maternal support, and mother–child relationship; (3) teacher–student relationship indicated by the four measuring items; and (4) student–student relationship indicated by the respective four measuring items. The three dimensions of character attribute (i.e., “treating others well,” “discipline and law-abidance,” and “traditional Chinese virtues.”) were three latent dependent variables indicated by the respective measuring items. Control variables were included in the model. The absolute model fit will be indicated by CFI (“Comparative Fit Index”), NNFI (“Non-Normed Fit Index”), RMSEA (“Root Mean Square Error of Approximation”), and SRMR (“Standardized Root Mean Square Residual”). Values above 0.90 for CFI and NNFI, together with a value below 0.08 for RMSEA and SRMR, suggest adequate model fit (55).

### Results

The structural model involving latent parental and school factors as predictors and latent character attributes in three dimensions as outcomes fitted the data well: χ^2^ = 3559.789, df = 819, χ^2^/df = 4.34, CFI = 0.92, NNFI = 0.92, RMSEA = 0.041, SRMR = 0.037. The standardized path coefficients were shown in Figure 1.

In addition to the above-hypothesized model including the main effects of parental and school factors, we also tested another model including both the main effects and the four interactions (paternal or maternal parenting × teacher–student or student–student relationship). As no significant interaction effects were identified (β ranged between −0.004 and 0.04, *p*s > 0.05), we focused on the main effects revealed in the hypothesized model in Figure 1.

Several observations can be highlighted from the present findings. First, as expected, both paternal and maternal positive parenting, as well as teacher–student and student–student relationships, significantly and positively predicted the three dimensions of character attributes, thus supporting Hypotheses 1a, 2a, and 2b. This model accounted for 18, 14, and 21% of the variance in the three dimensions, respectively. Second, further comparisons between path coefficients of paternal factor and maternal factor showed that maternal positive parenting (β = 0.24–0.26) had stronger predictions on adolescents' character attributes than did paternal positive parenting (β = 0.10–0.12, *z* = 2.85–3.90, *p*s < 0.01). In addition, while paternal parenting showed equivalent predictions on adolescents' character attributes as did the two school factors (|z| = 0.54–1.16, *p*s > 0.05), maternal parenting's effects were stronger than that of the two school factors (*z* = 2.27–5.22, *p*s < 0.05). Third, although the path coefficients of student–student relationship (β = 0.09–0.16) appeared to be stronger than that of teacher–student relationship (β = 0.06–0.09), the comparisons showed that the differences between the predictions of these two factors were not significant (*z* = 0.67–1.82, *p*s > 0.05).

Based on these findings, we concluded that a considerable proportion of variance in character attributes among adolescents are shaped by paternal and maternal positive parenting as well as teacher–student and student–student relationships. Among these factors, maternal positive parenting was the most salient one while others showed similar effects.

## Study 2: Findings Based on Parent Perspective

### Methods

#### Participants and Procedures

This study utilized parent data collected in the “Character building—A shared mission for a better future” project mentioned before. The data were collected in the “Parent Day” in nine schools when parents came to school to have a meeting with their children's class teachers to discuss the children's academic and behavioral performance in school (56). Biological parents were invited by the research team to complete a survey regarding the child studying in the school (if more than one child studies in the school, parents were instructed to focus on one randomly selected child). A total of 412 parents (mean age = 45.68, SD = 6.53) of Chinese adolescents (mean age = 14.67, SD = 1.85, 178 girls) signed written consent form and completed the survey. Among these parents, 76.0% (*n* = 313) were mothers. Most of the parents reported that they were in the first marriage (i.e., intact family, *n* = 326, 79.1%) or they did not encounter family economic disadvantages (*n* = 379, 92.0%). With reference to the latest figures released by the Hong Kong Council of Social Service (40) that 23.1% of children lived in poor families, the economically disadvantaged family may be relatively underrepresented in this study.

#### Measures

*Character Attributes* of adolescents were measured by the parent version of the 25-item PMCS mentioned in Study 1. In this study, parents evaluated their children's character attributes by reporting the extent to which their children possess each attribute on a 5-point scale (1 = “very much unlike my child,” 5 = “very much like my child”). The three-factor structure also fitted the data adequately. As shown in Table 2, Cronbach's α and McDonald's ω are above 0.85, suggesting adequate internal consistency of the three subscales.

**Table 2:** Descriptions and correlations among variables in Study 2 (parent data).

**Measures**	**Reliability (α/ω)**	***M***	***SD***	**Correlations**										
				**1**	**2**	**3**	**4**	**5**	**6**	**7**	**8**	**9**	**10**	**11**
Child age		14.67	1.85											
Child gender^a^				0.004										
Parent age		45.68	6.53	0.20^***^	−0.03									
Parent gender^a^				0.10	0.002	−0.30^***^								
FINT^b^				−0.04	0.03	−0.06	0.04							
FES^c^				0.06	−0.03	−0.14^**^	0.01	0.22^***^						
MCF1	0.89/0.88	4.14	0.54	0.03	0.11^*^	0.02	0.08	0.04	−0.12^*^					
MCF2	0.85/0.86	3.82	0.63	0.10^*^	0.12^*^	0.09	0.02	0.13^**^	−0.07	0.71^***^				
MCF3	0.87/0.87	3.89	0.64	0.10^*^	0.06	0.09	0.09	0.04	−0.06	0.72^***^	0.73^***^			
PBC	0.80/0.79	3.09	0.39	−0.07	−0.04	−0.04	0.13^**^	0.03	0.02	0.34^***^	0.28^***^	0.25^***^		
PPC	0.79/0.80	2.23	0.57	−0.05	0.002	−0.05	−0.04	0.04	−0.07	−0.04	−0.05	−0.07	0.10^*^	
PCR	0.83/0.83	3.06	0.56	−0.01	0.04	0.002	0.14^**^	0.10	0.01	0.31^***^	0.32^***^	0.27^***^	0.49^***^	−0.13^***^

*PBC, parental behavioral control; PPC, parental psychological control; PCR, parent–child relationship; FINT, family intactness; FES, family economic status; MCF1, Moral character factor 1-treating others well; MCF2, Moral character factor 2- self-discipline and law-abiding; MCF3, Moral character factor 3- traditional Chinese virtues*.

a *1 = male; 2 = female*.

b *1 = non-intact family; 2 = intact family*.

c *1 = having family economic disadvantage; 2 = without family economic disadvantage*.

* *p < 0.05;*

** *p < 0.01;*

*** *p < 0.001*.

*Parental Factors* were measured by the “Chinese Parent–Child Subsystem Quality Scale.” The original adolescent self-report version of this scale has been validated and widely used in previous studies involving Chinese adolescents (46, 57, 58). The present study employed a parent-report version where the parents were asked to rate their own parental practices on a 4-point scale (1 = “strongly disagree,” 4 = “strongly agree”). The scale included three subscales, including parental behavioral control (seven items, e.g., “I know my child's school life”), parental psychological control (four items, e.g., “I often want to change my child's mind or feelings for things”), and parent–child relationship (six items, e.g., “My child proactively shares his/her feelings with me”). The three-dimensional structure of the scale fitted the data adequately in the present study. Cronbach's α and McDonald's ω (i.e., 0.79–0.83) indicated adequate reliability of the three subscales in the present study (see Table 2).

*Covariates* included age and gender of the child, age, and gender of the parent who responded to the questionnaire, family intactness, and family economic status. The last two covariates were indicated by the same measures used in Study 1.

#### Data Analysis Plan

The data analysis procedure was similar to that in Study 1. We first checked CMB through Harman's single-factor test. We found that 28.55% of the variance in the measures of predictors and outcomes were explained by a single factor. This finding suggested that CMB was not a major issue in this study (54). Second, we checked multivariate normality of each item value for using ML in CFA (i.e., check the factor structures of character attributes and parental factors) and SEM (i.e., examine the association between parental factors and character attributes). In SEM, the three dimensions of character attributes of children were latent dependent variables while the three parental factors were latent predictors. The six covariates were controlled in SEM. Similar to Study 1, the model fit was assessed by CFI and NNFI (above 0.90) as well as RMSEA and SRMR (below 0.08).

### Results

**Figure 2:**
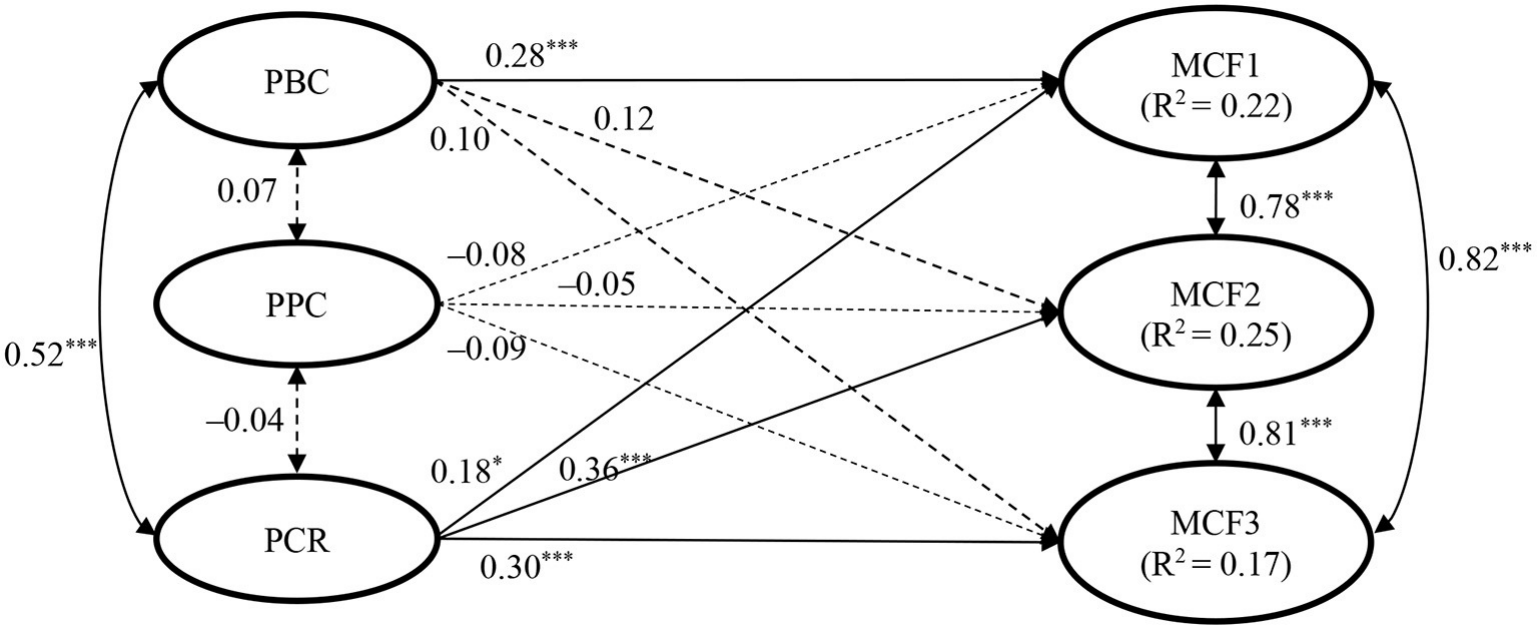
Standardized results of structural equation modeling on the relationships between parental factors and adolescent moral character in Study 2. Child age and gender, parent age and gender, family intactness, and family economic status were controlled. The control variables, observed indicators, and residuals were not shown in the figure. PBC, parental behavioral control; PPC, parental psychological control; PCR, parent–child relationship; MCF1, moral character factor 1 (i.e., treating others well); MCF2, moral character factor 2 (i.e., self-discipline and law-abidance); MCF3, moral character factor 3 (i.e., traditional Chinese virtues). χ^2^ = 1797.224, df = 955, χ^2^/df = 1.88, CFI = 0.868, NNFI = 0.858, RMSEA = 0.048, SRMR = 0.061. *p < 0.05; ***p < 0.001.

The structural model involving the three latent parental factors as predictors and the three dimensions of character attributes as outcomes fitted the data satisfactorily: χ^2^ = 1797.224, df = 955, χ^2^/df = 1.88, CFI = 0.87, NNFI = 0.86, RMSEA = 0.048, SRMR = 0.061. The standardized path coefficients were shown in Figure 2. The whole model explained 22, 25, and 17% of the variance in the three dimensions of character attributes, respectively.

Overall speaking, parental behavioral control and parent–child relationship as two positive parental factors positively predicted children's character attributes. More specifically, parental behavioral control positively predicted the character attributes of “treating others well” (i.e., the first dimension) among adolescents (β = 0.28, *p* < 0.001), but not the other two dimensions (“self-discipline and law-abidance” and “traditional Chinese virtues”). Another positive parental factor, parent–child relationship, positively predicted all the three dimensions of character attributes (β = 0.18–0.36, *p*s < 0.05). Thus, the results partially supported Hypothesis 1a from the parent perspective. In contrast to our expectation, parental psychological control as the negative parental factor did not show significant negative predictions on character attributes among adolescents. Thus, Hypothesis 1b was not supported from the parent perspective.

From the parent perspective, the findings of Study 2 provided additional support for the positive effects of positive parenting (e.g., behavioral control and good parent–child relationship) in the development of character attributes among adolescents. Furthermore, parents reported psychological control may not necessarily hinder children's character development.

## General Discussion

Developmental theories propose that children's character develops within the context of social interactions between the child and surrounding environments, such as parents, peers, and teachers (59, 60). Based on this notion, the present study focused on social influence on character development concerning two essential social contexts—family and school. Specifically, we examined how positive and negative parenting (parental factors), as well as teacher–student and student–student relationships (relational factors in school), shape adolescents' character attributes among Chinese secondary school students in Hong Kong. We used a survey design measuring parental factors, school factors, and adolescents' multiple character attributes from the perspectives of both adolescents and parents.

The results derived from child data support our hypothesis that both parents' positive parenting indexed by behavioral control, support, and a good relationship between parent and child has significant positive relationships with adolescents' character attributes. Findings based on the parent perspective also suggest positive impacts of behavioral control and a good parent–child relationship on children's character development. These findings echo Ngai's (32) and Karimi et al.'s (61) observations that parental care and authoritative parenting style positively predicted adolescents' character strengths such as kindness, love, self-regulation, and cooperativeness. Children are generally socialized by parents *via* parenting practices to think, act, and interact with others in certain ways. Accordingly, positive parenting behavior itself may serve as a behavioral guide for children to imitate and follow. For example, if children are cared for by parents, they may also show caring to others. Furthermore, warm and supportive parenting with firm regulations builds a nurturing environment that allows children to better understand the importance of values and norms and subsequently internalize moral codes and develop moral habits. For example, behavioral control and support promote self-regulation in daily conduct and open discussions about rules and responsibilities within a context of intimate emotional connections (62, 63). Taken together, positive parenting behaviors are constructive building blocks for character development among children (64, 65).

Findings also support the hypotheses that relational factors in school indexed by teacher–student and student–student relationships would positively predict character attributes among adolescents. As children grow up, the significance of secondary socialization agents, such as school, increases. It is commonly agreed that school experience mirrors shared norms, values, goals, and practices that shape youth development (35, 66). For example, teachers nurture a supportive context through listening to students' perspectives, offering feedback, providing choices for them to apply their strengths, and recognizing adolescents' achievement and good behaviors, which function as school-based ecological assets that help students develop internal models and strive for positive development in different domains, including character formation (35, 67). Likewise, through positive and effective peer interactions, students have a clear perception of peer expectations and are more intrinsically motivated to act in a prosocial manner and internalize prosocial values and moral codes (67, 68). The essential role played by supportive relationships in school in adolescents' character development has been incorporated into character education programs in that the establishment of a supportive school climate is central to program effectiveness (29, 69).

The above findings suggest that parents, teachers, and peers collectively shape adolescents' character development. A notable unique finding is that among the abovementioned predictors, maternal positive parenting showed the strongest effects on adolescents' character attributes. This indicates the salient role played by Chinese mothers in their children's development (49, 70), which may be explained by gender role differentiation in Chinese culture. Chinese mothers play a major role at home (e.g., cooking and looking after family members) while fathers are expected to provide financial support for the family, even though most Chinese mothers also have a job today. Mothers, in comparison to fathers, are more involved in children's lives in terms of spending more time and effort in communicating with, educating, and training children. Furthermore, Chinese mothers are expected to be loving while fathers are strict, as illustrated in the Chinese saying “yan fu ci mu” (“strict fathers and loving mothers”). Thus, it makes sense that maternal positive parenting creates an especially immediate nurturing environment for children to communicate moral issues, understand positive behaviors, and develop valued attributes (i.e., character attributes). However, this observation is only based on child data in Study 1. Besides, parent data in Study 2 did not control school factors, making it unable to examine parental influence in comparison to school impacts. Obviously, it will be meaningful to differentiate paternal and maternal parenting in parent data and explore the magnitude of their impacts while considering school factors as well.

Interestingly, different from our expectation, psychological control as an indicator of negative parenting did not negatively predict adolescents' character attributes. Some previous findings also suggest very weak or insignificant associations between parental psychological control and Chinese adolescents' developmental outcomes (49, 70). One possible explanation is that psychological control is not necessarily perceived as “negative” by Chinese adolescents. For example, Chinese children are more likely to interpret parents' controlling behavior as a way to express parental love and caring (71) and they also tend to perceive parents' psychological control as positively correlated with parental involvement (72). However, our finding and the proposed explanation are in contrast to a previous finding that higher levels of parental over-controlling behaviors (e.g., overprotection) predicted lower levels of character strengths, such as perseverance, kindness, and self-regulation, among Chinese adolescents (32). While Ngai's (32) study is based on child data, our finding is derived from parent data. The different findings might be related to the divergence between child ratings and parent ratings of parental behaviors and adolescents' character attributes (73, 74). Also, psychological control may be different from parental control which also includes elements of behavioral control. Nevertheless, the influence of parental psychological control on Chinese adolescents remains inconclusive and further research is needed to document the complex relationship between psychological control and children's character development from both child and parent perspectives.

The current findings have significant practical implications. The findings underscore parental impacts on the development of character attributes of their children. In particular, parental behavioral control, support, and good relationships with children lead to a nurturing family environment that positively contributes to adolescents' character development. This suggests that educators and professionals need to help parents reflect on their child-rearing behaviors and understand how their behaviors would impact their children. Parents can be educated to utilize more positive parenting strategies, such as behavioral control and being supportive, and to improve the relationship with their children. One promising way is to communicate more with children about related issues, such as parental expectation, the rationale of good character, and prosocial behavior. For example, recent research suggests that communication about moral issues such as fairness and justice in the family is conducive to character strength development among adolescents (75).

The influence of the two relational factors in school further suggests the importance of creating a favorable school environment for effective character education. To foster a positive teacher–student relationship, it is necessary to train teachers to demonstrate care and support through using different teaching strategies, tracking individual students' learning progress, providing timely evaluative feedback, showing interest in students' perspectives and personal problems, and engaging students in different extracurricular activities (35, 76). Meanwhile, it is also helpful to promote students' interpersonal relationships with both teachers and peers through skills training, such as social and emotional learning. It may be particularly relevant as social and emotional skills training is commonly overlooked in Chinese schools and Chinese adolescents have been found to lack such skills (77). Indeed, there are views advocating an integration between character education and social and emotional learning (21, 78). Given the essential roles played by parents and school, family–school cooperation is arguably indispensable in nurturing adolescents' character attributes. Literature also suggests that school character education would be more effective if there are home-based activities with parental involvement (79).

The present study has several limitations. First, although parental and school factors were treated as predictors of adolescents' character attributes, the inference of the causality is limited by the cross-sectional design. It is possible that adolescents' development also influences the surrounding contextual factors, such as parenting strategies and teacher–student relationship (49, 80–82). For example, adolescents' self-control (related to self-discipline) predicts subsequent parenting in a recent meta-analysis (81). Thus, future research needs to employ a longitudinal design and advanced analytical methods (e.g., cross-lagged panel analysis and parallel-process latent growth curve modeling) to further determine the direction of the relationship between positive institutions and character development among adolescents. For example, the examination of the possible bidirectional relationships between the contextual factor (e.g., parental or school factors) and adolescent character development would be an important area to be explored in future research.

Second, although we utilized child data and parent data and the findings on the predictions of parental factors on adolescent character attributes observed in the two studies collaborated to a certain extent, the data were collected through a single source (i.e., self-report data) in nature. Although self-report is regarded as a legitimate research method that is commonly used in youth studies [e.g., (63, 83, 84)], relying solely on adolescent self-assessment for both predictors and outcomes may lead to bias, particularly, common method biases (CMB). In the current study, we gave the participants clear instructions regarding anonymity and confidentiality and employed different reporting scale formats for predictors (4-point scale) and outcomes (5-point scale), which could help reduce CMB (54). In addition, Harman's single-factor tests suggested that CMB was not a major problem in the present study. Nevertheless, it is methodologically preferable to collect data from multiple informants (e.g., character attributes of the child being evaluated by parents) to examine possible differences between child-assessments and parent-assessments, which should be meaningful and interesting. For example, there are previous studies reporting differences between adolescents and parents regarding their assessments of parenting [e.g., (47, 73)]. Unfortunately, we were not able to match child and parent data in the present study. Future studies are needed to further examine the related child–parent discrepancy and how it may be related to children's developmental outcomes.

Third, although the interactions between parental and school factors were not the focus of the present study and the additional investigation in Study 1 did not show significant interactions, how the two socialization systems may interact with each other in shaping adolescents' character development deserves further exploration. In addition, as Study 2 only involved parental factors, future research should further control school factors (e.g., reported by children) when investigating parental influence using parent-report data. Fourth, while the sample in Study 1 showed characteristics similar to the general adolescent population, the family with economic disadvantages was underrepresented in Study 2, which may limit the generalizability of the current findings. More research is needed to determine the strength of associations between parental and school factors and youth character development in broader youth populations by including more diversified adolescent samples (and their parents), such as those from poor or single-parent families. Finally, the present study only involved Chinese adolescents in Hong Kong. It will be meaningful to replicate the present study in other Chinese communities, such as mainland China.

Despite the limitations, the present study provides empirical support for the influence of family and school on character development among Chinese adolescents based on data derived from both adolescents and parents. As expected, positive parental factors (behavioral control, support, and relationship between parent and child) and relational factors in school (teacher–student and student–student relationships) exert beneficial influences on adolescents' character attributes. Additionally, maternal positive parenting showed the strongest predictive effects. Furthermore, psychological control did not show hypothesized negative influence on adolescents' character attributes. These results highlight the importance of improving parenting strategies and relational factors in school through parent and teacher training.

## Data Availability Statement

The raw data supporting the conclusions of this article will be made available by the authors, without undue reservation.

## Ethics Statement

The studies involving human participants were reviewed and approved by Human Subjects Ethics Sub-Committee at the Hong Kong Polytechnic University. Written informed consent to participate in this study was provided by the participants' legal guardian/next of kin.

## Author Contributions

XZ contributed to the conceptualization, data interpretation, final analysis of the work, drafted the work, and revised it based on comments provided by DS. DS conceived the project and edited the manuscript. LY contributed to the design of the project and data collection. All authors contributed to the article and approved the submitted version.

## Funding

This work was supported by Wofoo Foundation and start-up grant to XZ (Project number: P0034745).

## Conflict of Interest

The authors declare that the research was conducted in the absence of any commercial or financial relationships that could be construed as a potential conflict of interest.

## Publisher's Note

All claims expressed in this article are solely those of the authors and do not necessarily represent those of their affiliated organizations, or those of the publisher, the editors and the reviewers. Any product that may be evaluated in this article, or claim that may be made by its manufacturer, is not guaranteed or endorsed by the publisher.

## References

[1] Polanczyk GV Salum GA Sugaya LS Caye A Rohde LA. Annual research review: a meta-analysis of the worldwide prevalence of mental disorders in children and adolescents. J Child Psychol Psychiatry. (2015) 56:345–65. 10.1111/jcpp.12381 25649325

[2] World Health Organization. Adolescent Mental Health. (2019). Available online at: https://www.who.int/news-room/fact-sheets/detail/adolescent-mental-health (accessed September 20, 2021).

[3] Bruha L Spyridou V Forth G Ougrin D. Global child and adolescent mental health: challenges and advances. London J Primary Care. (2018) 10:108–9. 10.1080/17571472.2018.1484332

[4] Seligman MEP Csikszentmihalyi M. Positive psychology: an introduction. In: Flow and the Foundations of Positive Psychology. Cham: Springer (2014). p. 279–98. 10.1007/978-94-017-9088-8_18

[5] Duan W Bai Y Tang X Siu PY Ho SM. Virtues and positive mental health. Hong Kong Journal of Mental Health. (2012) 38:24–31.

[6] Peterson C Seligman ME. Character Strengths and Virtues: A Handbook and Classification. Oxford: Oxford University Press (2004).

[7] Ruch W Wagner L. Character building and adolescent development. In: Hupp S Jewell JD editors. The Encyclopedia of Child and Adolescent Development. Vol. 7. New York, NY: Wiley (2020). p. 3243–56. 10.1002/9781119171492.wecad333

[8] Shek DTL Zhu X. Self-reported risk and delinquent behavior and problem behavioral intention in Hong Kong adolescents: the role of moral competence and spirituality. Front Psychol. (2018) 9:430. 10.3389/fpsyg.2018.00430 29651269 PMC5885157

[9] Wagner L Ruch W. Good character at school: positive classroom behavior mediates the link between character strengths and school achievement. Front Psychol. (2015) 6:610. 10.3389/fpsyg.2015.00610 26029144 PMC4432234

[10] Zhou Z Shek DTL Zhu X Lin L. The influence of moral character attributes on adolescent life satisfaction: the mediating role of responsible behavior. Child Indic Res. (2021) 14:1293–313. 10.1007/s12187-020-09797-7

[11] Cloninger CR Svrakic DM Przybeck TR. A psychobiological model of temperament and character. Arch Gen Psychiatry. (1993) 50:975–90. 10.1001/archpsyc.1993.01820240059008 8250684

[12] Friedman HS Schustack MW. Personality: Classic Theories and Modern Research (6th ed.). London: Pearson Education (2016).

[13] Bajraktarov S Novotni A Arsova S Gudeva-Nikovska D Vujovik V. (2017). Character and temperament dimensions in subjects with depressive disorder: impact of the affective state on their expression. Open Access Macedonian J Med Sci 5:64–7. 10.3889/oamjms.2017.012 28293319 PMC5320910

[14] Sperandeo R Maldonato M Baldo G Dell'Orco S. Executive functions, temperament and character traits: A quantitative analysis of the relationship between personality and prefrontal functions. In: 7th IEEE International Conference on Cognitive Infocommunications (CogInfoCom). Wroclaw (2016). 10.1109/CogInfoCom.2016.7804522

[15] Aquino K Reed A. The self-importance of moral identity. J Pers Soc Psychol. (2002) 83:1423–40. 10.1037/0022-3514.83.6.1423 12500822

[16] Hardy SA Walker LJ Olsen JA Woodbury RD Hickman JR. Moral identity as moral ideal self: Links to adolescent outcomes. Dev Psychol. (2014) 50:45–57. 10.1037/a0033598 23895167

[17] Huta V Ryan RM. Pursuing pleasure or virtue: The differential and overlapping well-being benefits of hedonic and eudaimonic motives. J Happiness Stud. (2010) 11:735–62. 10.1007/s10902-009-9171-4

[18] Lerner RM Castellino DR. Contemporary developmental theory and adolescence: Developmental systems and applied developmental science. J Adolesc Health. (2002) 31:122–35. 10.1016/S1054-139X(02)00495-0 12470909

[19] Lerner RM Callina KS. The study of character development: Towards tests of a relational developmental systems model. Human Dev. (2014) 57:322–46. 10.1159/000368784

[20] Narvaez D. Human flourishing and moral development: Cognitive and neurobiological perspectives of virtue development. In: Nucci LP Narvaez D editors. Handbook of Moral and Character Education (1st ed.). Oxford: Routledge (2008). p. 310−27.

[21] Narvaez D Lapsley D. Becoming a moral person – Moral development and moral character education as a result of social interactions. In: Christen M van Schaik CP Fischer J Huppenbauer M Tanner C editors. Empirically Informed Ethics: Morality Between Facts and Norms. Switzerland: Springer (2014). p. 227–38. 10.1007/978-3-319-01369-5_13

[22] Park N. The role of subjective well-being in positive youth development. Ann Am Acad Polit Soc Sci. (2004) 591:25–39. 10.1177/0002716203260078

[23] Hands CM. Circles of influence: The role of school-community partnerships in the character formation and citizenship of secondary school students. Alberta J Educ Res. (2008) 54:50–64. 10.11575/ajer.v54i1.55210

[24] Kochanska G. Mutually responsive orientation between mothers and their young children: A context for the early development of conscience. Curr Dir Psychol Sci. (2002) 11:191–5. 10.1111/1467-8721.00198

[25] Grégoire J Pauwels LJR. Do specific combinations of parent–child relationships predict moral values? Dev Behav. (2020) 41:1485–509. 10.1080/01639625.2019.1627018

[26] Karmakar R. (2015). Does parenting style influence the internalization of moral values in children and adolescents? Psychol Stud. 60:438–46. 10.1007/s12646-015-0338-2

[27] World Health Organization. Health for the World's Adolescents: A Second Chance in the Second Decade. (2014). Available online at: https://apps.who.int/adolescent/second-decade/ (accessed September 20, 2021).

[28] Berkowitz MW Bier MC. What Works in Character Education*: A Research-Driven Guide for Educators*. Character Education Partnership. Washington, DC: Character Education Partnership (2005).

[29] Marshall JC Caldwell SD Foster J. Moral education the CHARACTER plus Way®. J Moral Educ. (2011) 40:51–72. 10.1080/03057240.2011.541770

[30] Narvaez D Bock T. Developing ethical expertise and moral personalities. In: Nucci L Narvaez D Krettenauer T editors. Handbook of Moral Character Education (2nd ed.). Routledge (2014). p. 140–58.

[31] Peterson C. A Primer in Positive Psychology. Oxford: Oxford University Press (2006).

[32] Ngai SSY. Parental bonding and character strengths among Chinese adolescents in Hong Kong. Int J Adolesc Youth. (2015) 20:317–33. 10.1080/02673843.2015.1007879

[33] NgaiSSY Cheung CK Xie L NgYH Ngai HL Liu Y. Psychometric properties of the parental bonding instrument: Data from a Chinese adolescent sample in Hong Kong. J Child Family Stud. (2018) 27:2112–24. 10.1007/s10826-018-1058-8

[34] Raimundi MJ Molina MF Schmidt V Hernández-Mendo A. Family functioning profiles and character strengths in young elite athletes from Argentina. Int J Sport Exerc Psychol. (2019) 17:32–51. 10.1080/1612197X.2016.1189949

[35] Shubert J. Developmental change in character strengths across childhood and adolescence and in school contexts (Dissertation), University of Rochester, Rochester, NY (2018).

[36] Carretero CM Gimeno EMC. Testing a predictive model of school climate on character development and bullying behaviors. Estudios Sobre Educ. (2019) 37:135–57. 10.15581/004.37.135-157

[37] Shek DTL Lee BM Yu L Lin L Ma CMS Wu FKY. Character-building education for children and adolescents in Hong Kong: What can we learn from research? Int J Child Adolesc Health. (2021) 14:225–39.

[38] Shek DTL Lin L. A Study on the Development of Chinese Students (Character, Psychosocial Competence and Behavior): Report No. 1 – Views of Students. Hong Kong: Department of Applied Social Sciences, The Hong Kong Polytechnic University (2017).

[39] Chan YC Leung MC Tsang KMS Lu H Fok HK. Report on Parenting Practices in Hong Kong. Family Council (2017). Available online at: https://www.familycouncil.gov.hk/en/files/research/Final%20report_study_on_parenting_practices_in_hong_kong.pdf (accessed January 13, 2022).

[40] The Hong Kong Council of Social Service. Percentage of Children Aged 0-14 in Low-Income Domestic Households Among All Persons Aged 0-14 in Domestic Households. Retrieved from: https://www.socialindicators.org.hk/en/indicators/children/29.8 (accessed January 13, 2022).

[41] Wan Z Yang S. Qing shao nian dao de zi wo ren tong wen juan de xiu ding [The revision of moral self-identity scale for Chinese adolescents]. Sci Social Psychol. (2008) 5:41–6.

[42] Chen L. Characters of adolescents' values and related research (Masther thesis), Shanghai Normal University, Shanghai (2008).

[43] Yang H Stoeber J Wang Y. Moral perfectionism and moral values, virtues, and judgments: a preliminary investigation. Pers Ind Diff . (2015) 75:229–33. 10.1016/j.paid.2014.11.040

[44] Schwartz SH. Draft Users Manual: Proper Use of the Schwarz Value Survey, Version 14. Centre for Cross Cultural Comparisons (2009). Available online at: http://www.crossculturalcentre.homestead.com (accessed October 20, 2021).

[45] Shek DTL. Perceived parental control processes, parent-child relational qualities, and psychological well-being in Chinese adolescents with and without economic disadvantage. J Genet Psychol. (2005) 166:171–88. 10.3200/GNTP.166.2.171-188 15906930

[46] Shek DTL Law MYM. Dimensionality of the Chinese parent-child subsystem quality scale: confirmatory factor analyses. Int J Child Health Human Dev. (2016) 9:207–15.

[47] Leung JTY Shek DTL. Parent–adolescent discrepancies in perceived parenting characteristics and adolescent developmental outcomes in poor Chinese families. J Child Family Stud. (2014) 23:200–13. 10.1007/s10826-013-9775-5 24482569 PMC3890555

[48] Shek DTL Zhu X. Paternal and maternal influence on delinquency among early adolescents in Hong Kong. Int J Environ Res Public Health. (2019) 16:1338. 10.3390/ijerph16081338 31013967 PMC6518268

[49] Zhu X Shek DTL. The influence of adolescent problem behaviors on life satisfaction: Parent–child subsystem qualities as mediators. Child Indic Res. (2020) 13:1767–89. 10.1007/s12187-020-09719-7

[50] Teng Z Bear GG Yang C Nie Q Guo C. Moral disengagement and bullying perpetration: A longitudinal study of the moderating effect of school climate. School Psychol. (2020) 35:99–109. 10.1037/spq0000348 31804101

[51] Xie J-S Lv Y-X Ma K Xie L. Te La Hua Xiao Yuan Fen Wei Liang Biao (Xue Sheng Juan) Zhong Wen Ban Xin Xiao Du Yan Jiu [Reliability and validity of the Chinese version of Delaware School Climate survey–student]. Chin J Clin Psychol. (2016) 24:250–3. 10.16128/j.cnki.1005-3611.2016.02.014

[52] Finney SJ DiStefano C. Non-normal and categorical data in structural equation modeling. In: Hancock GR Mueller RO editors. Structural Equation Modeling: A Second Course. Greenwich, CT: Information Age Publishing (2006). p. 269–314.

[53] Heck RH Thomas SL Tabata LN. Multilevel and Longitudinal Modeling With IBM SPSS (2nd ed.). New York, NY: Routledge (2014). 10.4324/9780203701249

[54] Podsakoff PM MacKenzie SB Lee JY Podsakoff NP. Common method biases in behavioral research: a critical review of the literature and recommended remedies. J Appl Psychol. (2003) 88:879–903. 10.1037/0021-9010.88.5.879 14516251

[55] Kline RB. Principles and Practice of Structural Equation Modeling (Fourth ed.). (2015). New York, NY: The Guilford Press.

[56] Shek DTL Yu L. A Study on the Development of Chinese Students (Character, Psychosocial Competence and Behavior): Report No. 3 – Views of Parents. Department of Applied Social Sciences, The Hong Kong Polytechnic University (2018).

[57] Leung JTY Shek DTL. Parental control and adolescent wellbeing in Chinese adolescents in Hong Kong. Child Indic Res. (2020) 13:703–27. 10.1007/s12187-019-09642-6

[58] Zhu X Shek DTL. Parental factors and adolescent well-being: Associations between developmental trajectories. Child Youth Serv Rev. (2021) 127:106071. 10.1016/j.childyouth.2021.106071

[59] Clement S Bollinger R. Perspectives on character virtue development. Res Human Dev. (2016) 13:174–81. 10.1080/15427609.2016.1172445

[60] Wang J Batanova M Ferris KA Lerner RM. Character development within the relational developmental systems metatheory: a view of the issues. Res Human Dev. (2016) 13:91–6. 10.1080/15427609.2016.1165932

[61] Karimi S Pirmoradi M Ashouri A Aghebati A. Relationship of temperament and character traits, emotional regulation and perceived parenting with self-harming behaviors in adolescents. Iran J Psychiatry Clin Psychol. (2021) 26:448–63. 10.32598/ijpcp.26.4.2955.2.1

[62] Barber BK Stolz HE Olsen JA Collins WA Burchinal M. Parental support, psychological control, and behavioral control: Assessing relevance across time, culture, and method. Monogr Soc Res Child Dev. (2005) 70:26–57. 10.1111/j.1540-5834.2005.00368.x 16359423

[63] Kaniušonyte G Laursen B. Parenting styles revisited: a longitudinal person-oriented assessment of perceived parent behavior. J Social Pers Relat. (2021) 38:210–31. 10.1177/0265407520960818

[64] Ramos AM Griffin AM Neiderhiser JM Reiss D. Did I inherit my moral compass? Examining socialization and evocative mechanisms for virtuous character development. Behav Genet. (2019) 49:175–86. 10.1007/s10519-018-09945-4 30656439 PMC6443408

[65] Tu YC Chou MJ Lee HC. Paren-child shared reading meets information technology: Revealing links between parenting and children's character development. J Appl Sci. (2013) 13:1029–36. 10.3923/jas.2013.1029.1036

[66] Cohen J McCabe L Michelli NM Pickeral T. School climate: research, policy, practice, and teacher education. Teach Coll Rec. (2009) 111:180–213. 10.1177/016146810911100108

[67] Wentzel KR Muenks K McNeish D Russell S. Peer and teacher supports in relation to motivation and effort: a multi-level study. Contemp Educ Psychol. (2017) 49:32–45. 10.1016/j.cedpsych.2016.11.002

[68] Pavey L Greitemeyer T Sparks P. Highlighting relatedness promotes prosocial motives and behavior. Pers Social Psychol Bull. (2011) 37:905–17. 10.1177/0146167211405994 21521720

[69] Agboola A Tsai KC. Bring character education into classroom. Euro J Educ Res. (2012) 1:163–70. 10.12973/eu-jer.1.2.163

[70] Xia YR Wang C Li W Wilson S Bush KR Peterson G. Chinese parenting behaviors, adolescent school adjustment, and problem behavior. Marriage Family Rev. (2015) 51:489–515. 10.1080/01494929.2015.10384089120405

[71] Cheung CSS McBride-Chang C. Relations of perceived maternal parenting style, practices, and learning motivation to academic competence in Chinese children. Merrill-Palmer Q. (2008) 54:1–22. 10.1353/mpq.2008.0011

[72] Cheung CSS Pomerantz EM. Parents' involvement in children's learning in the United States and China: implications for children's academic and emotional adjustment. Child Dev. (2011) 82:932–50. 10.1111/j.1467-8624.2011.01582.x 21418057 PMC3089668

[73] Mastrotheodoros S Van der Graaff J Dekovi ć M Meeus WHJ Branje SJT. Coming closer in adolescence: Convergence in mother, father, and adolescent reports of parenting. J Res Adolesc. (2019) 29:846–62. 10.1111/jora.12417 29921030 PMC6899895

[74] Ruch W Weber M Park N Peterson C. Character strengths in children and adolescents. Euro J Psychol Assess. (2014) 30:57–64. 10.1027/1015-5759/a000169

[75] Shubert J Wray-Lake L Syvertsen AK Metzger A. The role of family civic context in character development across childhood and adolescence. Appl Dev Sci. (2019) 26:15–30. 10.1080/10888691.2019.1683452

[76] Suldo SM Friedrich AA White T Farmer J Minch D Michalowski J. Teacher support and adolescents' subjective well-being: a mixed-methods investigation. School Psychol Rev. (2009) 38:67–85. 10.1080/02796015.2009.12087850

[77] Shek DTL Lin L Ma CMS Yu L Leung JTY Wu FKY. Perceptions of adolescents, teachers and parents of life skills education and life skills in high school students in Hong Kong. Appl Res Qual Life. (2020) 16:1847–60. 10.1007/s11482-020-09848-9

[78] Elias MJ. Social-emotional and character development and academics as a dual focus of educational policy. Educ Policy. (2009) 23:831–46. 10.1177/0895904808330167

[79] Paul SAS Hart P Augustin L Clarke PJ Pike M. Parents' perspectives on home-based character education activities. J Fam Stud. (2020) 10.1080/13229400.2020.1806097

[80] Li JB Bi SS Willems YE Finkenauer C. The association between school discipline and self-control from preschoolers to high school students: a three-level meta-analysis. Rev Educ Res. (2021) 91:73–111. 10.3102/0034654320979160

[81] Li JB Willems YE Stok FM Deković M Bartels M Finkenauer C. Parenting and self-control across early to late adolescence: A three-level meta-analysis. Persp Psychol Sci. (2019) 14:967–1005. 10.1177/1745691619863046 31491364

[82] Zhu X Shek DTL. Parental control and adolescent delinquency based on parallel process latent growth curve modeling. Int J Environ Res Public Health. (2021) 18:8916. 10.3390/ijerph18178916 34501506 PMC8431368

[83] Fang Q Liu C Tang Y Shi Z Wang Q Helwig CC. Types of parental psychological control and rural and urban Chinese adolescents' psychological well-being and academic functioning. Child Dev. (2021). 10.1111/cdev.13699 34729781

[84] Suldo SM Thalji-Raitano A Hasemeyer M Gelley CD Hoy B. Understanding middle school students life satisfaction: does school climate matter? Appl Res Qual Life. (2013) 8:169–82. 10.1007/s11482-012-9185-7

